# Helicobacter Pylori Infection in Endoscopic Biopsy Specimens of Gastric Cancer: A Preliminary Evaluation in a High Risk Population of Kashmir Valley

**DOI:** 10.1155/DTE.4.35

**Published:** 1997

**Authors:** G. M. Malik, S. Kadla, M. Mubarik, T. Hussain, G. Jeelani, J. Basu, S. Rashid, M. Ahmad, A. Nafee, A. R. Khan

**Affiliations:** 1 Department of Gastroenterology SMHS Hospital Government Medical College Srinagar Kashmir India; 2 Department of Medicine SMHS Hospital Government Medical College Srinagar Kashmir India; 3 Department of Pathology SMHS Hospital Government Medical College Srinagar Kashmir India; 4 R/O. Nalband-pora Safakadal Srinagar Kashmir 190 002 India

## Abstract

*Objective*: The aim of the present study was to assess the prevalence of Helicobacter pylori (H. pylori) infection in Kashmiri patients with gastric cancer and to compare this with a matched control population.

*Methods*: Fifty patients with gastric cancer and thirty age/sex matched controls were included in the study. All the subjects were hailing from Kashmir Valley. For detection of H. pylori, biopsy specimens were used both from cases and controls.

*Results*: An insignificant association was shown between H. pylori and both intestinal and diffuse type of gastric cancer.

*Conclusions*: The data provides support against the significant association between H. pylori and gastric cancer in this part of world, a place where the age standardized incidence of gastric cancer is alarmingly high. We conclude that other factors like personal and special dietary habits of Kashmiri population may be more important for the development of gastric cancer.


*Diagnostic and Therapeutic Endoscopy*, Vol. 4, pp. 35–42
Reprints available directly from the publisher
Photocopying permitted by license only

© 1997 OPA (Overseas Publishers Association)
Amsterdam B.V. Published in The Netherlands
by Harwood Academic Publishers
Printed in Singapore

### Helicobacter Pylori Infection in Endoscopic Biopsy Specimens of Gastric Cancer: A Preliminary Evaluation in a High Risk Population of Kashmir Valley

G.M. MALIK[a], S. KADLA[a], M. MUBARIK[a,*], T. HUSSAIN[a], G. JEELANI[a], J. BASU[a], S. RASHID[a], M. AHMAD[a], A. NAFEE[b] and A.R. KHAN[c]

[a]*Department of Gastroenterology;* [b]*Department of Medicine;* [c]*Department of Pathology; SMHS Hospital, Government Medical College, Srinagar, Kashmir, India*

*(Received 19 August 1996; In final form 24 January 1997)*

***Objective*: The aim of the present study was to assess the prevalence of Helicobacter pylori (H. pylori) infection in Kashmiri patients with gastric cancer and to compare this with a matched control population.**

***Methods*: Fifty patients with gastric cancer and thirty age/sex matched controls were included in the study. All the subjects were hailing from Kashmir Valley. For detection of H. pylori, biopsy specimens were used both from cases and controls.**

***Results*: An insignificant association was shown between H. pylori and both intestinal and diffuse type of gastric cancer.**

***Conclusions*: The data provides support against the significant association between H. pylori and gastric cancer in this part of world, a place where the age standardized incidence of gastric cancer is alarmingly high. We conclude that other factors like personal and special dietary habits of Kashmiri population may be more important for the development of gastric cancer.**

*Keywords:* Helicobacter pylori, Prevalence, Gastric, Cancer, Biopsy

### INTRODUCTION

Recently population based studies have revealed an alarmingly high incidence of gastric cancer in Kashmir (males 36.7/100,000/year, females 9.7/100,000/year) [1]. Little is known about the factors responsible for this. Studies from other countries have revealed an increased risk of gastric cancer due to infection with H. pylori. Such a study has not been conducted in Kashmir Valley as yet. The present study was undertaken to have a preliminary viewpoint regarding the association between gastric cancer and H. pylori in this valley.

The mechanisms involved in the gastric carcinogenosis due to infection with H. pylori are not fully understood, but recently a model of the

* Corresponding author. Also at: R/O. Nalband-pora, Safakadal, Srinagar – 190 002, Kashmir (India).

chronological changes that occur during the development of gastric cancer has been proposed. According to this, H. pylori infection leads to chronic inflammation of gastric epithelial cells due to which these cells get damaged at DNA level which in turn leads to mutation which ultimately culminates in gastric atrophy and intestinal metaplasia and hence gastric cancer [2]. Studies have suggested that the organism is strongly associated with gastric cancer and lymphomas of mucosa associated lymphatic tissue (MALT) [3].

Evidence supporting a role of H. pylori in genesis of gastric cancer has come from three well designed seroepidemiological studies [4–6]. These studies reported that persons who have been infected with H. pylori upto 25 years previously had a significantly higher risk of later developing gastric cancer than those persons who at that time had not been infected by H. pylori (mean time between serum collection and diagnosis of gastric cancer ranged from 6 to 14.2 years). In these studies, the Odd's ratio of persons infected with H. pylori developing gastric cancer ranged from 2.77 to 9.5.

The Eurogast study group concluded six fold increase in the risk of gastric cancer in populations with 100% H. pylori infection compared with populations that have no infection [7]. Notwithstanding, the results of studies examining the point prevalence of H. pylori in gastric cancer patients have varied significantly with some studies showing the prevalence of this organism in gastric cancer patients to be similar to or lower than that in control population [8–11].

The aims of the present study were multifold. The first was to have a preliminary assessment regarding the prevalence of H. pylori in patients with gastric cancer, living in Kashmir Valley, a place with a high incidence of this cancer. Second we compared the prevalence of H. pylori infection in the patient population with an age/sex matched control population. Third, we examined the association of H. pylori with both intestinal and diffuse types of gastric cancer and finally we attempted to examine the association of H. pylori with tumours occurring in the region of gastric antrum and with those occurring in the regions other than gastric antrum.

### MATERIAL AND METHODS

(i) *Cases*: The cases comprised 50 patients with gastric cancer. Their mean age $\pm$ SD was $55.30 \pm 8.86$ years. Of these 50 patients, 42(84%) were males and 08(16%) were females. There was no significant difference in age between male and female cases. These subjects were selected from the patients referred for endoscopic examination from the hospitals affiliated to Government Medical College, Srinagar. All the subjects were interviewed regarding drug intake (antibiotics, $H_2$ blockers and colloidal bismuth) one month prior to endoscopic biopsy. Patients, who had such a history were excluded from the study. Patients who had gastroesophageal junction tumours were also excluded from the study. The diagnosis of the gastric cancer was confirmed by histopathological examination of biopsy material after processing. During upper G.I. endoscopy, the type of gastric growth viz. polypoid, ulcerative, infiltrative and fungating growth, the rough size of growth and location of growth either in the corpus or the fundus, or the antrum were also recorded.

(ii) *Controls*: The controls comprised 30 healthy volunteers who were apparently free of any disease and had no history of drug intake (antibiotics, $H_2$ blockers and colloidal bismuth) one month prior to endoscopy. They were matched for both age and sex and all of them were hailing from the valley.

(iii) *Endoscopy and biopsy specimens*: All the cases and controls underwent upper G.I. endoscopy using injection diazepam 10 mgs I/V as premedication. The cope used was GIF/GQ-Olympus. Once growth was seen in cases, multiple (at least two) biopsy specimens were collected from each of the following sites: (1) within the cancer itself, (2) the paracancerous area 2 cms distal to the growth, (3) the antrum – in those patients who had growth away from this site and (4) the corpus and the fundus in those patients who had antral growth [13].

In controls, multiple biopsies were taken from the antrum.

The biopsy specimens were subjected to following test procedures:

(a) *One Minute Endoscopy Room Test (OMERT)*: In this test, two biopsy specimens were put in one ml of 10% W/V freshly prepared urea solution in deionized water (pH 6.8) at room temperature. Two drops of 1% phenol red were added to above solution as an indicator. A change in colour from yellow to pink observed 1–5 minutes after addition of indicator was taken as positive test (i.e. H. pylori present), whereas absence of such colour change or change of colour after 5 minutes was taken as negative test [14].

(b) *Histology*: Biopsy specimens were fixed in 10% buffered formalin and were processed routinely [10,15,16]. Paraffin sections (5 µm) were cut and stained with: (1) hematoxylin and eosin and (2) May–Graunwald–Giemsa stain [13]. The sections were studied for histopathological features of gastric mucosa (gastritis, metaplastic and mitotic changes) as well as for the presence of organisms of H. pylori. Gastric cancer was classified as intestinal or diffuse by the classification system of Lauren [17].

(c) *Gram's staining*: Two biopsy specimens from each region were rubbed on glass slides (separate glass slides used for separate site), heat fixed and then stained with Gram's stain and were then studied for presence of H. pylori under the light microscope [18].

The result obtained, i.e. demonstration of H. pylori in the biopsy specimen stained by May–Graunwald–Giemsa stain, was taken as gold standard as per the current recommendations [3,12,15,16,19].

(d) *Statistical analysis*: For statistical analysis the chi-square test was applied. A $p$-value of less than 0.05 was considered significant.

(e) *Ethics*: All subjects gave informed consent for the collection of biopsy tissue. Human experimentation guidelines of the "Declaration of Helsinki" were followed and the study was approved by Principal/Dean, Government Medical College, Srinagar, after consideration and approval by members of "Board of Studies".

### RESULTS

*Prevalence of H. pylori infection in gastric cancer patients and in controls*: Helicobacterpylori positivity among cases was 17(34%) by histology, 17(34%) by Gram's staining and 16(32%) by one minute endoscopy room test (Table I). Two cases who came positive by histology were not detected by OMERT and Gram's staining. However, the results from histology were taken as gold standard [3].

Helicobacter pylori positivity among controls was 10(33.33%) by histology, 9(30%) by Gram's staining and 8(26.67%) by OMERT (Table II). There was one individual among controls who was positive for H. pylori by histology but negative by Gram's staining and OMERT.

*Location of the tumour and H. pylori positivity*: Among 50 cases, 26(52%) had growth around the antral region called antral growth and 24(48%)

TABLE I Helicobacter positivity among cases ($n = 50$)

| | Test | No. of cases | No. of +ve cases | %age |
|---|---|---|---|---|
| 01 | Histology | 50 | 17 | 34.00 |
| 02 | Gram's Staining | 50 | 17 | 34.00 |
| 03 | One minute endoscopy room test (OMERT) | 50 | 16 | 32.00 |

cases had growth at the regions other than antrum called non-antral growth. Out of 26 cases with antral growth, 11(42.31%) were positive for H. pylori and among rest 24 cases, 6(25%) were positive for H. pylori by histology (Table III).

*Histological typing of the gastric cancer and H. pylori status*: Among 50 cases, 33(66%) patients had intestinal type of carcinoma stomach, out of them 11(33.33%) were positive for H. pylori. The rest 17(34%) cases had diffuse type of carcinoma stomach of whom only 6(35.29%) were positive for H. pylori (Table IV).

*Helicobacter pylori positivity in relation with biopsy site*: Examination of tissue taken from different biopsy sites in histologically H. pylori positive gastric cancer patients, has shown organisms to be present in only 10% of the biopsies taken from cancer area, while the positivity was more in the biopsies taken from paracancerous area (50%). The positivity was 62.8% from fundal biopsies and 80.2% for antral biopsies. The overall bacterial load was high with antral biopsies.

*Size of tumour and H. pylori positivity*: The prevalence of H. pylori infection in relation to tumour size was also studied. Among 50 cases, 38(76%) had tumours more than 5 cms in size and 12(24%) had tumours less than 5 cms. The H. pylori positivity in former group was 9(23.68%) and 8(66.66%) in the latter group. This is a significantly high rate of infection in individuals with small tumours compared to those with large tumours of more than 5 cms in size.

*Endoscopic type of gastric cancer and H. pylori positivity*: Endoscopically, the growths were divided in infiltrative, ulcerative, fungating and polypoid type of growths. The number of such tumours types and their H. pylori status is revealed in Table V.

*Gastric mucosal histology and H. pylori positivity*: Of 50 cases, the histology of the biopsy

TABLE II Helicobacter positivity among controls ($n = 30$)

| | Test | No. of cases | No. of +ve cases | % age |
|---|---|---|---|---|
| 01 | Histology | 30 | 10 | 33.33 |
| 02 | Gram's Staining | 30 | 09 | 30.00 |
| 03 | One minute endoscopy room test (OMERT) | 30 | 08 | 26.67 |

TABLE III Helicobacter pylori positivity with respect to type of growth among cases ($n = 50$)

| Type of growth | No. (% age) | H. pylori positivity by histology No. (% age) |
|---|---|---|
| Antral growths | 26 (52.00) | 11 (42.31) |
| Non-antral growths | 24 (48.00) | 06 (25.00) |

TABLE IV Helicobacter pylori positivity with reference to Histological type of growth among cases ($n = 50$)

| Histological type of growth | No. (% age) | H. pylori positivity by histology No. (% age) |
|---|---|---|
| Intestinal type | 33 (66.00) | 11 (33.33) |
| Diffuse type | 17 (34.00) | 06 (35.29) |

TABLE V H. pylori positivity in relation to endoscopic type of tumour ($n = 50$)

| Type of tumour | No. of cases No. (%) | H. pylori Positivity by | | |
|---|---|---|---|---|
| | | Histology No. (%) | Gram's staining No. (%) | OMERT No. (%) |
| Infiltrative | 30 (60.00) | 12 (40.00) | 12 (40.00) | 10 (33.33) |
| Ulcerative | 16 (32.00) | 04 (25.00) | 04 (25.00) | 05 (31.25) |
| Polypoid | 04 (8.00) | 01 (25.00) | 01 (25.00) | 01 (25.00) |
| Total: | 50 | 17 (34.00) | 17 (34.00) | 16 (32.00) |

OMERT = One minute Endoscopy room test, Statistical Remarks: $X^2 df_2 = 1.14$, (Using histology as gold standard) $p > 0.50$, Insignificant.

TABLE VI H. pylori positivity in relation to histological finding of macroscopically normal looking gastric mucosa among cases ($n = 50$)

| Histology | No. of cases No. (%) | H. pylori Positivity by | | |
|---|---|---|---|---|
| | | Histology No. (%) | Gram's staining No. (%) | OMERT No. (%) |
| Normal gastric mucosa | 46 (92.00) | 14 (30.43) | 14 (30.43) | 13 (32.51) |
| Chronic active gastritis | 03 (6.00) | 02 (66.66) | 02 (66.66) | 02 (66.66) |
| Chronic superficial gastritis | 01 (2.00) | 01 (100.00) | 01 (100.00) | 01 (100.00) |
| Total: | 50 | 17 (34.00) | 17 (34.00) | 16 (32.00) |

Statistical Remarks: $X^2 df_2 = 3.63$ (Using histology) $p > 0.10$, Insignificant.
Note: None of our patients had chronic atrophic gastritis or intestinal metaplasia.

TABLE VII H. pylori positivity in relation to histological findings among controls ($n = 30$)

| Histology | No. of cases No. (%) | H. pylori Positivity by | | |
|---|---|---|---|---|
| | | Histology No. (%) | Gram's staining No. (%) | OMERT No. (%) |
| Normal gastric mucosa | 20 (66.66) | 05 (25.00) | 03 (15.00) | 03 (15.00) |
| Chronic superficial gastritis | 06 (20.00) | 04 (66.66) | 05 (83.33) | 04 (66.66) |
| Chronic active gastritis | 04 (13.33) | 01 (25.00) | 01 (25.00) | 01 (25.00) |
| Total: | 30 | 10 (33.33) | 09 (30.00) | 08 (26.66) |

Statistical Remarks: $X^2 df_2 = 3.75$ (Using histology) $p > 0.10$, Insignificant.
Note: None of our controls had chronic atrophic gastritis or intestinal metaplasia.

specimen taken at the sites other than cancer site revealed features as depicted in Table VI.

Similarly among 30 controls, the histopathology of biopsy revealed features as depicted in Table VII.

Comparison of the prevalence rate of H. pylori infection in gastric cancer patients and controls.

The H. pylori positivity between cases and controls was compared statistically. The $X^2 df_1$ value equaled 0.003 and $p$ value $> 0.90$ which means an insignificant association between H. pylori and carcinoma stomach (Table VIII).

The H. pylori positivity and its association to gastric cancer with respect to type of tumour and site of tumour are depicted in Tables IX and X.

### DISCUSSION

Association between Helicobacter pylori infection and gastric cancer has been supported by a

TABLE VIII Relationship between Helicobacter pylori and carcinoma stomach

| Group | No. of cases | Helicobacter positivity by histology No. (% age) | Statistical inference |
|---|---|---|---|
| Cases | 50 | 17 (34.00) | $X^2df_1 = 0.003, p > 0.90$ |
| Controls | 30 | 10 (33.33) | Insignificant |

TABLE IX Relationship between H. pylori and antral growths of stomach

| Group | No. of cases | H. pylori positivity by histology No. (% age) | Statistical inference |
|---|---|---|---|
| Antral growths | 26 | 11 (42.31) | $X^2df_1 = 0.62, p > 0.25$ |
| Controls | 30 | 10 (33.33) | Insignificant |

TABLE X Relationship between H. pylori and intestinal type of carcinoma stomach

| Group | No. of cases | H. pylori positivity by histology No. (% age) | Statistical inference |
|---|---|---|---|
| Cases of intestinal type of carcinoma | 33 | 11 (33.33) | No variation in incidence rates |
| Controls | 30 | 10 (33.33) | Insignificant |

universal phenomenon and until there exists a satisfactory explanation of the mechanism by which H. pylori influences gastric carcinogenesis, a causal link cannot be assumed. The Eurogast study group [7] has found out 6 fold increased risk of gastric cancer in populations with 100% H. pylori infection compared with populations that have no infection. However, there are places where despite the fact that the prevalence of H. pylori infection has been shown to be high, there is a low incidence of gastric cancer [20, 21].

The valley of Kashmir in the Indian subcontinent is a high incidence area of gastric cancer [1]. The prevalence of H. pylori in the normal population has been found to be 33.33% by histology, whereas the present study has shown that in this area those who developed gastric cancer had no significant increase in the prevalence of H. pylori infection. This implies that mere infection with H. pylori cannot explain such a high incidence of gastric cancer in this part of world.

The patients who had gastroesophageal junction growths were excluded from the study because these growths frequently arise from the abnormal mucosa in Barett's esophagus and therefore cannot be ascribed to H. pylori infection and hence the confounding effect of Barrett's esophagus was removed [6]. Another group of patients who were excluded from the study were those who had taken antibiotics, $H_2$ blockers and colloidal bismuth in the month prior to endoscopic examination because the histological results become less reliable marker of H. pylori infection [12].

Our study is different from the studies which support the association between H. pylori and carcinoma stomach. Such studies have been undertaken in different parts of world and most of these studies have used ELISA method for detection of antibodies against H. pylori. They have also used different stains for histology (Giemsa, methylene blue) for detecting H. pylori. The results obtained from these studies have shown that by ELISA serology, the results of H. pylori positivity have been very high as compared to those found by histology. In other words we can say that histology underestimates the H. pylori infectivity. One of the reasons given is that the sensitivity and the specificity of ELISA are less as compared to histology. Second reason is that the chances of finding H. pylori in the biopsy specimens by histology become less when the changes of chronic atrophic gastritis and intestinal metaplasia are set in the stomach [22]. These changes although being early steps in the evolution of gastric cancer in H. pylori infected patients, yet lead to absence or decrease in the H. pylori load in gastritis and metaplastic changed areas of the stomach. This also explains why the organisms of H. pylori are less frequently detected from biopsy specimens of the cancerous site. The reason for such a quantitative change is not known but it has been postulated that the organism loses its ecological niche in the stomach once the atrophic gastritis and metaplastic changes occur [23].

In our study, none of the cases had above mentioned pre-cancerous changes, therefore we believe that detection of H. pylori by histology has not underestimated the H. pylori infectivity results as has been reported in other studies [13].

It has been thought that the initiation and promotion of the pre-cancerous lesions i.e. chronic atrophic gastritis and intestinal metaplasia, by H. pylori are influenced by several factors including gastric hypoacidity, bacterial overgrowth and diets low in anti-oxidants but high in irritants and mutagen precursors [2]. Helicobacter pylori infection among cases and controls almost in equal percentages (Cases 34% and Controls 33.33%) in this short study rules out this infection to be a single causal agent for cancer stomach. But we believe that the high incidence of gastric cancer in Kashmir Valley could also be due to other factors like diet which is peculiar among Kashmiri people. Such diets include excessive consumpion of salty tea, pickles, dry fruit, dried vegetables and leaves of Brassica Olericeaea (Hak) [1].

In conclusion, the present study does not show any significant association between H. pylori infection and gastric cancer in high risk population of Kashmir Valley. The association is insignificant for both the types of Leuren's group of gastric cancer. Besides present study also concludes an insignificant association between antral tumours and H. pylori infectivity in Kashmir Valley. However, further studies especially on a larger group of patients, are needed to draw any conclusion in this regard.

***Acknowledgement***

We are indebted to Mr. Emm Afsurdah for his hard-work in preparing this manuscript.

***References***

[1] Khuroo, M.S., Zargar, S.A., Mahajan, R., *et al.* High incidence of esophageal and gastric cancer in Kashmir – in a population with special personal and dietary habits. *Gut* 1992; **33**: 11–5.

[2] Julie Parsonnet. H. pylori and Gastric Cancer. *Gastroenterology Clinics of North America* 1993; **22**(1): 89–104.

[3] Barry, J. and Marshell, M.D. Helicobacter pylori. *Amm. Jr. Gatro. Suppl.* 1994; **89**(8): 116–28.

[4] Forman, D., Newwell, D.G., Fullerton, F., *et al.* Association between infection with Helicobacter pylori and risk of gastric cancer: evidence from a prospective investigation. *BMJ* 1991; **302**: 1302–05.

[5] Abraham Nomura, Grant, N., Stemmermann, Pro-Huang Chyou, *et al.* Helicobacter pylori infection and gastric carcinoma among Japanese Americans in Hawaii. *The NEGM* 1991; **325**(16): 1132–4.

[6] Julie Parsonnet, Gary, D., Friedman, *et al.* Helicobacter pylori infection and the risk of gastric carcinoma. *The New England Journal of Medicine* 1991; **325**(16): 1127–31.

[7] Eurogast study group. An international association between Helicobacter pylori and gastric cancer. *Lancet* 1993; **341**(8857): 1359–62.

[8] Yong Yi Feng and Wang, Y.A. Campylobacter pylori in patients with gastritis, peptic ulcer and carcinoma of the stomach in Lanzhou, China. *The Lancet* 1988; p. 1055–56.

[9] Clarkson, K.S. and West, K.P. Gastric cancer and H. pylori infection. *J. Clin. Pathol.* 1993; **46**: 997–9.

[10] Loffeld, R.J.L.F., Willems, I., Flendrig, J.A., *et al.* Helicobacter pylori and gastric carcinoma. *Histopathology* 1990; **17**: 537.

[11] Kuipers, E.J., Graciacasanova, M., Pena, A.S., *et al.* H. pylori and gastric carcinoma, *Scand. J. Gastroenterol.* 1993; **28**: 433–7.

[12] Logan, R.P.H., Polson, R.J., Misiewicz, J.J., *et al.* Simplified single sample $^{13}$Carbon urea breath test for helicobacter pylori. Comparison with histology, culture, and ELISA Serology, *Gut* 1991; **32**: 1461–6.

[13] HU, P.J., Mitchll, H.M., Li, Y.Y., *et al.* Association of H. pylori with gastric cancer and observations on the detection of this bacterium in gastric cancer cases. *The Am. Jr. of Gastroenterol.* 1994; **89**(10): 1806–10.

[14] Arvind, A.S., Cook, R.S., Tabaq, S., *et al.* One minute endoscopy room test for campylobacter pylori. *Lancet* 1988; p. 704.

[15] Abdelrahman Elshiekh Mohammed, Mohammed Ali Al-Karawi, Abdelrahman Abdullah Al-Jumah, *et al.* Helicobacter pylori: Prevalence in 352 consecutive patients with Dyspepsia. *Annals of Saudi Medicine*, 1994; **2**: 134–5.

[16] Mohammed A.E., Al-Karawi, A., Ahmed A.M.M, *et al.* Helicobacter pylori: Incidence and comparison of three diagnostic methods in 196 Saudi patients with dyspepsia. *Hepato-Gastroenterol.*, 1994; **41**: 48–50.

[17] Lauren, P. The two histological main types of gastric carcinoma. Diffuse and so called intestinal type carcinoma – An attempt at a histo-clinical classification. *Acta Pathol. Microbiol. Scand.* 1965; p. 31–49.

[18] Jiang, S.J., Liu, W.Z., Zhang, D.Z., *et al.* Campylobacter like organisms in chronic gastritis, peptic ulcer, and gastric carcinoma. *Scand. J. Gastroenterol.* 1987; **22**: 553–8.

[19] Robey-Cafferty, S.S., Ro, J.Y. and Clear, K.R. The prevalence of Campylobacter pylori in gastric biopsies from cancer patients. *Mod. Pathol.* 1989; **2**: 473.

[20] Mitchell, H.M., Li, Y.Y., HU, P.J., *et al.* Epidemiology of H. pylori in Southern China: Identification of early childhood as the critical period of acquisition. *J. Infect. Dis.* 1992; **166**: 149–53.

[21] Mitchell, H.M. The epidemiology of H. pylori infection and its relation to gastric cancer. In: Goodwin C.S., Wormslay B.W., eds. H. pylori – *Biology and Clinical Practices*, Florida: CRC Press 1993; pp. 95–114.

[22] Sipponen, P., Kosunen, T.U., Valle, J., *et al.* H. pylori infection and chronic gastitision gastric cancer. *J. Clin. Pathol.* 1992; **45**: 319–23.

[23] Siurala, M., Sipponen, P. and Kekki, M. Chronic gastritis. Dynamic and Clinical aspects. *Scand. J. Gastroenterol. Suppl.* 1985; **109**: 69.

